# Trust your gut: using physiological states as a source of information is almost as effective as optimal Bayesian learning

**DOI:** 10.1098/rspb.2017.2411

**Published:** 2018-01-24

**Authors:** Andrew D. Higginson, Tim W. Fawcett, Alasdair I. Houston, John M. McNamara

**Affiliations:** 1 Centre for Research in Animal Behaviour, College of Life and Environmental Sciences, University of Exeter, Exeter EX4 4QG, UK; 2 School of Biological Sciences, Life Sciences Building, Tyndall Avenue, Bristol BS8 1TQ, UK; 3 School of Mathematics, University of Bristol, University Walk, Bristol BS8 1TW, UK

**Keywords:** behavioural gambit, cognition, computational costs, decision-making, information use, optimal foraging

## Abstract

Approaches to understanding adaptive behaviour often assume that animals have perfect information about environmental conditions or are capable of sophisticated learning. If such learning abilities are costly, however, natural selection will favour simpler mechanisms for controlling behaviour when faced with uncertain conditions. Here, we show that, in a foraging context, a strategy based only on current energy reserves often performs almost as well as a Bayesian learning strategy that integrates all previous experiences to form an optimal estimate of environmental conditions. We find that Bayesian learning gives a strong advantage only if fluctuations in the food supply are very strong and reasonably frequent. The performance of both the Bayesian and the reserve-based strategy are more robust to inaccurate knowledge of the temporal pattern of environmental conditions than a strategy that has perfect knowledge about current conditions. Studies assuming Bayesian learning are often accused of being unrealistic; our results suggest that animals can achieve a similar level of performance to Bayesians using much simpler mechanisms based on their physiological state. More broadly, our work suggests that the ability to use internal states as a source of information about recent environmental conditions will have weakened selection for sophisticated learning and decision-making systems.

## 1. Introduction

‘Il meglio è nemico del bene' [‘The best is enemy of the good’]

Italian proverb

The study of animal decision-making has typically taken an optimization approach in which the animal is assumed to have perfect knowledge of current and long-term conditions [1–4]. In reality, animals will be uncertain about conditions [5]. Such uncertainty can be incorporated into evolutionary models using Bayes's rule, which updates knowledge given new information in a logically consistent way [6,7], invoking the behavioural gambit [8] that animals will behave *as though* they can perform Bayesian calculations [9,10]. However, it remains unclear how most animals could approximate Bayesian learning without invoking implausible computational abilities or excessively costly physiological or cognitive mechanisms that would require a large brain. For a mechanism to be favoured by selection, there needs to be sufficient advantage to the animal in terms of reproductive success to offset the costs of the mechanism. In many situations, a simpler but less accurate mechanism, determining a ‘rule of thumb' or heuristic, might be advantageous if it has a smaller cost [11]. An example is simple learning rules based on a linear operator [12]. Such rules may also be more robust than Bayesian learning, in that their performance is less affected if information is imperfect [12,13].

One of the best-studied situations in decision-making is searching for food [4,14,15]. Described rules of thumb include the ‘two-strikes’ rule that bees (*Bombus lapidarius*) appear to follow in making patch-quitting decisions [16]; the animal acts as though it has a fixed memory window for foraging success, such as remembering whether or not it found food on the last few occasions that it looked. Another example is the constant time in patches used by caddis fly larvae (*Plectrocnemia conspersa*) [17]; here the animal acts as though it keeps track of time and ignores changes in conditions. Both methods may lead to behaviour that is similar to a more sophisticated system that tracks food availability explicitly [16,17].

To behave optimally in different conditions, the animal needs some way of assessing current conditions. In the case of foraging, the animal discovers food items stochastically, which does not necessarily reflect the overall food abundance at that point in time. Animals therefore need some way to integrate past events, but acquiring and processing information in a Bayesian way is likely to be costly [18]. Instead, natural selection could exploit the fact that animals have internal states that are a potential source of information about conditions. All else being equal, energetic reserves tend to increase if food is abundant and fall if food is scarce. Since conditions are positively autocorrelated over time in most natural environments, conditions in the recent past are informative of current conditions [5]. As such, reserves could act as a physiological ‘memory' of environmental conditions and so indicate current conditions [19].

Here, we show that energy reserves, a physiological state, provides a simple yet surprisingly effective cue to decide how intensively to forage for food. For clarity, we use a simple model of survival in a fluctuating environment (i.e. the generalized risk allocation model of [20]), where food availability varies over time. We characterize the animal's environment in terms of the distribution, variability and abundance of food items. We investigate under what conditions we expect animals to behave as though they have sophisticated learning mechanisms for assessing current conditions, when they should have simpler mechanisms and when they should ignore fluctuations in conditions altogether. To predict the outcome of natural selection it would be necessary to quantify the cost of mental mechanisms, but this is currently not possible. We therefore compare the survivorship of various candidate mechanisms to understand when sophisticated mechanisms give large benefits, in which case animals are unlikely to have simple mechanisms. We find that, across a wide range of situations, a strategy based only on the level of reserves performs almost as well as optimal Bayesian learning, despite being much simpler, because reserve level acts as a memory. We discuss how such mechanisms may operate in non-foraging contexts too, and suggest that physiological states acting as ‘memories' may be ubiquitous.

## 2. The model

We are interested in the foraging strategy that maximizes survival in a temporally changing environment where death can occur through starvation or predation. One possible response to harsh conditions is to cease activity and wait for better times, but the consequences of this for the forager's survival and future state will depend on its current reserves. We therefore use a state-dependent model in which the optimal action is allowed to depend on both the current conditions and the current level of reserves. We model behaviour over a long sequence of discrete time steps. The animal and its environment are characterized by two states: its level of reserves *x* (*x* ≥ 0) and the current environmental conditions *E* where food availability is higher in good conditions (*E = G*) than bad conditions (*E = B*). Food availability differs only in the maximum probability of finding food when foraging (*γ*_G_ and *γ*_B_, where *γ*_G_ ≥ *γ*_B_).

The food availability of the environment is assumed to fluctuate over time. Incorporating environmental heterogeneity into models of adaptive behaviour requires the inclusion of an environmental state variable [5]. Often we can capture sufficient complexity with just two possible environmental states A and B, such as high and low food availability. Next, we characterize stochastic transitions between the two environmental states. The simplest case is where the probability of transition (per unit time) between states depends only on the current state. At the end of a time step, we assume that the environment changes from the current conditions *E* to the alternative conditions with probability *λ_E_*. Thus, a good environment becomes a bad environment with probability *λ*_G_, while a bad environment becomes a good environment with probability *λ*_B_. The duration of both good and bad periods follow a geometric distribution whose mean is the reciprocal of the transition probabilities, which we term *t*_G_ and *t*_B_, respectively. Note that this environment will show positive temporal autocorrelation if *λ*_B_ + *λ*_G_ < 1 because then conditions are more likely to stay the same than to change [5].

The aspect of behaviour we are interested in is foraging intensity, which we call *f* (0 ≤ *f* ≤ 1). Increasing *f* increases the probability of finding food but also increases exposure to predators and hence the probability of being attacked. We assume that while the animal is not foraging, it is safe from predation. We also assume that predation risk when foraging increases with energy reserves *x* because of decreasing manoeuvrability [21]. (Regardless of the exact cost, some cost needs to be assumed if long-term adaptive fat levels are to be stable [22].) In a given time step, the probability of mortality of the animal due to predation (*μ*) is given by2.1
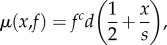
where *c* controls how the risk increases with *f*, *d* is the maximum probability of predator attack, and *s* is the maximum reserve level. We assume that the forager uses *m* units of energy per time step on metabolism and finds a food item with probability *γ_E_f.* For computational reasons, there is some variance in the energy content of food items (see electronic supplementary material [hereafter ESM], appendix): food items contain either *b*_1_ or *b*_2_ units of energy; for the results shown in the main text, we assume that items with energy *b*_1_ = 5 and *b*_2_ = 6 occur with equal probability. The reserves at the next time step are therefore
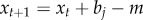
after a successful discovery of food item of type *j* (*j* = 1,2), and
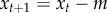
after a failure to find food. If *x_t_* = 0, then *x_t_*_+1_ = 0 because the animal is dead.

A strategy specifies how *f* depends on circumstances (e.g. reserves, information). We find optimal strategies of various classes, all of which minimize the mortality rate and so maximize the survival probability over a long time period. The classes of strategy differ in the constraints on the information available to the forager. Where the current environmental state *E* is known (perfect information), this is the generalized risk allocation model [20]. Where *E* is not known, the forager may be able to estimate it based on available cues. To model this, we include a state variable *ρ* to represent the forager's estimated probability that conditions are currently good (i.e. that *E* = *G*). Here we find the optimal strategy f* from two classes of strategy in which information is imperfect: (i) the animal estimates the probability *ρ* that conditions are currently good directly from its foraging experiences, using Bayesian updating; (ii) the animal does not monitor its foraging experiences directly but is sensitive to its current energy reserves, and can take into account the fact that the level of reserves is informative of recent conditions to estimate *ρ*. Assuming that the forager is optimally adapted to minimize its long-term mortality rate, we use dynamic programming to find optimal solutions given the constraints on information (see ESM, appendix A). We set other parameter values (*m, d, c, b_j_*) such that the risk of mortality over some long time period is realistic. If each time step is thought of as around 1 h, then 2000 time steps represent around 100 days of winter, over which the animals try to survive. Small birds in temperate regions survive winter with 50–70% probability [23–25], so we tune the parameters such that the survival at the baseline parameter values is around this range. As mortality is far from both zero and one, this ensures that the model can make clear predictions about the effects of the parameter values of interest on the performance of the various strategies.

We compare the performance of these constrained optimal strategies to two other classes of strategy that would be optimal if the environmental conditions were unchanging:

(1) A ‘pessimistic' class of strategy that behaves as though the food availability is constantly low (*γ*_B_). (We do not show results for the alternative ‘optimistic' strategy that behaves as though food availability is constantly high (*γ*_G_), because it performs very poorly in all non-trivial conditions.)
(2) An optimally biased strategy that behaves as though the food availability is high with a fixed probability and low otherwise, where the fixed probability is that which is optimal, and so will have been naturally selected for in the absence of any attempt to track food availability.

Thus, in summary we compare the performance of five classes of strategy:

— **Perfect (*P*):** Forager has perfect knowledge about current food availability.
— **Bayesian (*L*):** Forager uses Bayes's theorem to estimate current food availability directly from its foraging experiences.
— **Reserves (*R*):** Forager does not monitor its foraging experiences but can base its decisions on its current reserve level; note that, through natural selection, the response to reserves will be influenced by the conditional probability that food availability is high given the reserve level.
— **Pessimist (*S*):** Forager behaves as though the current food availability is always low.
— **Optimal bias (*U*)**: Forager behaves as though the current food availability is high with a fixed probability ρ*, which is the estimate that minimizes the long-term mortality rate.

For each class, we find the optimal foraging strategy as a function of reserves and information state. We then assess the resulting survival over 2000 time steps starting from the stationary distribution of *x* in the population. To do this, we simulate a population following the optimal strategy until the distribution of individuals stops changing, rescale so the size of the population is unity, and then run for 2000 time steps to determine the survival probability *Q*(*i*), where *i* indicates one of the strategy classes as shown above. All parameters and their baseline values are shown in table 1.

**Table 1.** Parameters in the model and their default values.

symbol	description	value
*s*	maximum level of reserves	100
*m*	energy use per unit time	1
*b_j_*	energy in food item type *j*	5, 6
*d*	maximum probability of predator attack	0.002
*c*	power of relationship between foraging and predation risk	2
*ψ*	survival cost per time step for reserve-based strategies	0.001, 0.004
*k*	relative cost of Bayesian compared with reserve-based strategy	2
*γ_E_*	probability of finding food per unit time spent foraging in environment in condition *E*	*γ*_G_ = 0.7, *γ*_B_ = 0.3
*λ_E_*	probability that environment in condition *E* changes to the other condition	*λ*_G_ = 0.01, *λ*_B_ = 0.01
*t_E_*	mean number of time steps for which environment stays in condition *E* (*t_E_* = 1/*λ_E_*)	*t*_G_ = 100, *t*_B_ = 100

## 3. Results

**Figure 1.**
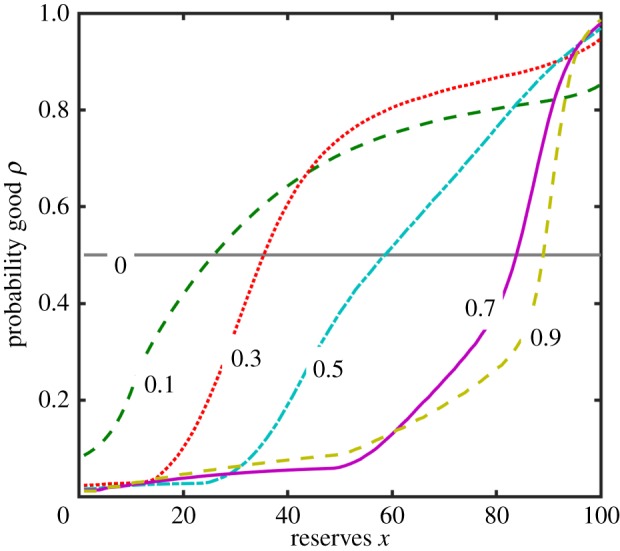
Probability that conditions are good given current reserves *x* under the reserve-based strategy *R, ρ*(*G|x*). The values on each line indicate the difference between conditions in food availability *γ*_G_ − *γ*_B_, where *γ*_G_ + *γ*_B_ = 1. Mean duration of bad and good periods: *t*_B_ = *t*_G_ = 0.005. (Online version in colour.)

When using the reserve-based strategy (class *R*) the probability that conditions are good as a function of reserves *x* is shown in figure 1. For all parameter settings, the probability follows a sigmoid curve, with a low probability that conditions are good at low reserves and a high probability at high reserves, because reserves gradually build up when food is abundant and decrease when food is scarce. The curve shifts to the right as the difference between *γ*_G_ and *γ*_B_ increases, because the optimal strategy is to store more reserves in good conditions to prepare for bad conditions. The steepness of the sigmoid curve depends on the fluctuation rate (ESM, figure B1).

**Figure 2.**
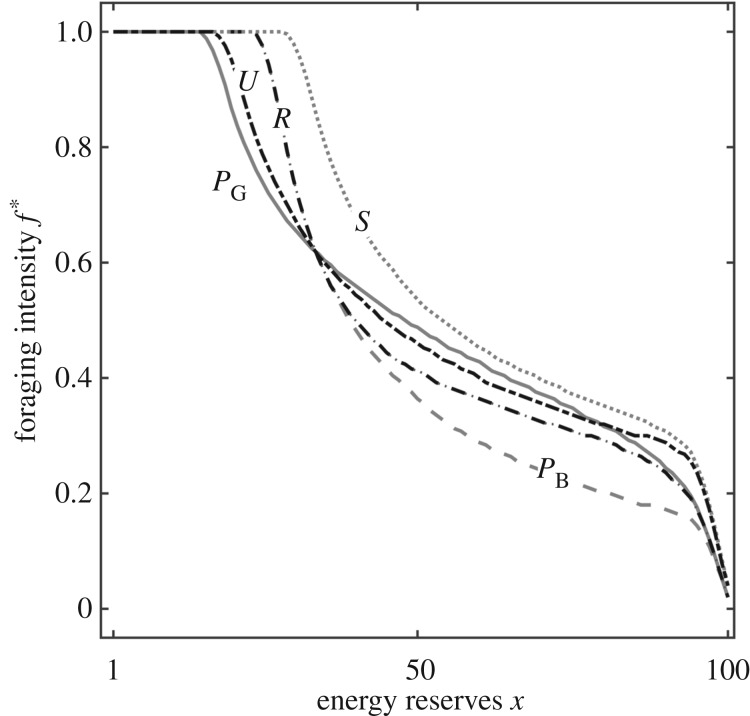
The optimal foraging intensity (f*) as a function of energy reserves (*x*) for the perfect information (grey lines: *P*_G_ solid, *P*_B_ dashed), reserve-based (*R*), pessimistic (*S*) and optimal bias (*U*) cases. For the optimal Bayesian learning strategy (*L*), the values of *f*_L_* are intermediate to those for *P*_G_ and *P*_B_ (shown in ESM, figure B3).

The optimal foraging intensity f* for all five strategy classes is shown in figure 2 for the baseline parameter values (with the differences in foraging intensity plotted in ESM, figure B2). As we have shown previously [20], there is a crossover point in the optimal intensity of foraging under perfect information f*_P_ (grey lines), with more intense foraging when food availability is low if reserves are low [f*_P_(*x,B*) > f*_P_(*x,G*) when *x* < 30], but less intense foraging when food availability is low if reserves are high [f*_P_(*x,B*) < f*_P_(*x,G*) when *x* ≥ 30]. A pessimist has f*_S_ that is too high because it does not expect good conditions to occur at all. For the reserve-based optimal strategy, foraging intensity f*_R_ is similar to f*_P_(*x,B*) when reserves are low and closer to f*_P_(*x,G*) when reserves are high (compare grey and dotted lines). This is intuitive, because the lower the reserve level, the more likely it is that conditions are bad, hence the animal should behave as though conditions are bad; whereas if reserves are high it is likely that conditions are good, hence the animal should behave as though conditions are good. For the Bayesian learning strategy, f*_L_ is similar to f*_P_(*x*,*B*) when the posterior probability that conditions are currently good *ρ* is zero and similar to f*_P_(*x*,*G*) when *ρ* is unity, with a gradual change in f*_L_ for intermediate *ρ* (ESM, figure B3).

**Figure 3.**
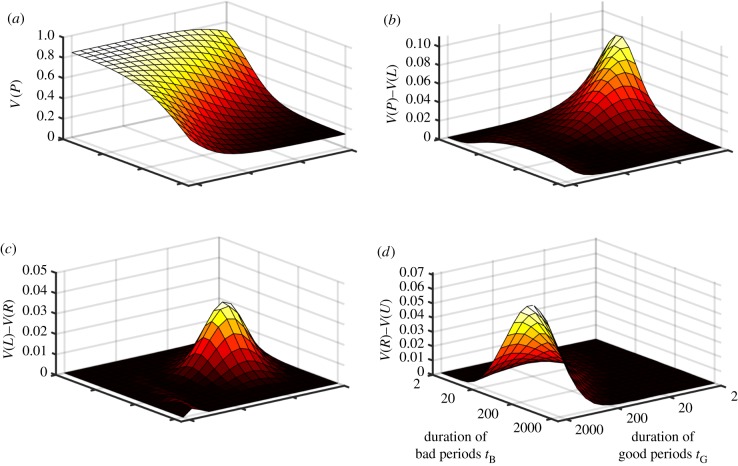
Comparison of survival probabilities over 2000 time steps *Q(i)* for the various methods across parameter space (*t*_B_ and *t*_G_ shown on axes). We show results for the baseline probability of finding food in the two conditions (*γ*_B_ = 0.25, *γ*_G_ = 0.75); for other values, see ESM, figure B4. (*a*) Survival under perfect information (*P*)*.* (*b*–*d*) Differences in survival between strategies (*b*) *P* and *L* (Bayesian learner), (*c*) *L* and *R* (reserve-based), and (*d*) *R* and *U* (optimal bias). Note the different scales of the vertical axes. (Online version in colour.)

We assess the probability of surviving 2000 time steps for each optimal strategy under various conditions (figure 3; shown for *γ*_B_ = 0.25 and *γ*_G_ = 0.75, for other values see ESM, figure B4). For clarity, we first show survival under perfect knowledge (*P*, which always does best) and then the differences between the various strategies. Survival always increases with the mean duration of good periods and decreases with the mean duration of bad periods because mortality mostly occurs in bad periods, and the length of these therefore determines survival (figure 3*a*; ESM, figure B4a–e). Survival decreases as the difference in food availability increases because that determines the severity of bad periods, except that survival increases with the difference in food availability if conditions are good most of the time (cf. ESM, figure B4a,d), because the increased rate of gain in good periods more than compensates for this and risk allocation has a large benefit.

In general, the difference in survival between perfect knowledge (*P*) and the information-constrained strategies (*L, R*) is much less than 5% for most conditions. *L* (Bayesian learning) does worst compared to *P* when periods are short because it is impossible to learn fast enough to perform risk allocation effectively (figure 3*b*; ESM, figure B4f–j); this is exacerbated when food availability differs markedly between good and bad conditions (ESM, figure B4j). Across parameter space, there is strikingly little difference between *L* and the reserve-based strategy *R* (figure 3*c*; ESM, figure B4 k–o), except when periods are moderately short (around 20 time steps) and the difference in food availability between conditions is very large (ESM, figure B4o). *R* does much better than *U* (optimal bias) when periods are long and of roughly equal duration, because then it is most important to do the correct thing (figure 3*d*; ESM, figure B4p–t). The optimal estimate ρ* under the *U* strategy is always smaller than the actual *ρ* (ESM, figure B5). This is because eating too much in good conditions is less deleterious than eating too little in poor conditions.

**Figure 4.**
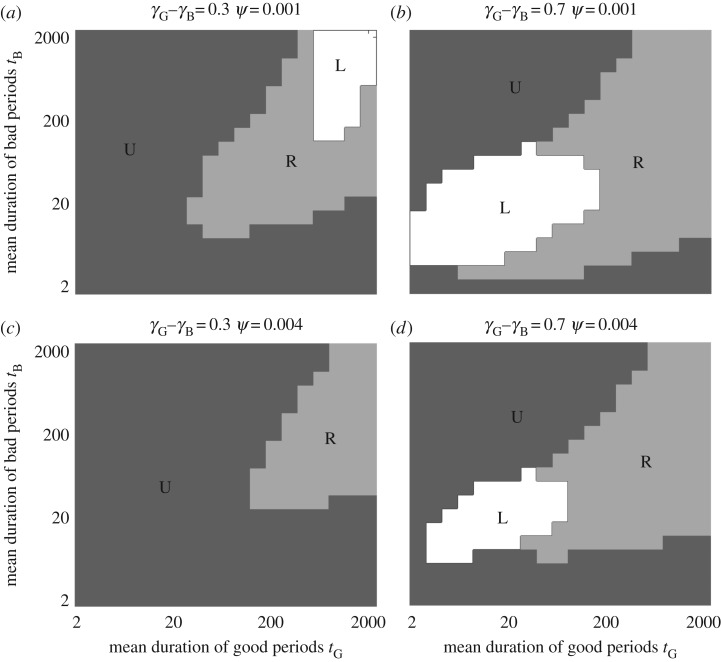
Best strategy class under varying costs of implementation. For comparison, we assumed that the reserve-based (*R*) strategy pays an arbitrary survival cost *ψ* per time step whereas the Bayesian learning (*L*) strategy pays *kψ*; the optimal bias (*U*) and pessimistic (*S*) strategies pay no cost. We assume that the *P* strategy cannot evolve. The shaded regions indicate which strategy (*L, R* or *U*) has highest survival given these costs for (*a*,*b*) *ψ* = 0.001 and (*c*,*d*) *ψ* = 0.004, and for (*a*,*c*) *γ*_B_ = 0.35, *γ*_G_ = 0.65 and (*b*,*d*) *γ*_B_ = 0.15, *γ*_G_ = 0.85, with *k* = 2 in all panels. The results do not qualitatively depend on the values of *ψ* or *k*, with an intuitive gradual shrinking of the *L* region as *k* (relative cost of Bayesian learning compared to a reserve-based strategy) increases.

In figure 4, we clarify the conditions under which a learning (*L*) or reserve-based (*R*) strategy should evolve, under the arbitrary assumption that *L* is twice as costly as *R*. We expect sophisticated learning to be worth this additional cost when periods are moderately short and food availability changes greatly (bottom-left of figure 4*b*,*d*) or when the fluctuations are subtle and infrequent (top-right of figure 4*a*). We expect the reserve-based strategy to be favoured if the world is not predominantly poor or rich (i.e. along the main diagonal of figure 4) and does not change too quickly (not the bottom-left). This is because *R* does not adapt fast enough when conditions turn bad and so the animal is more likely to die; in this situation, either *L* or *U* does better. In all other cases, decisions based solely on the current reserve level allow the animal to perform almost as well as a sophisticated Bayesian learning strategy, with differences less than 1% in most of parameter space, and 0.04% for the baseline parameter values.

For the results above, we assumed that *γ*_G_ + *γ*_B_ = 1. However, the difference between *L* and *R* remains small for almost all combinations of *γ*_G_ and *γ*_B_ (ESM, figure B6). We have also confirmed that the results are not sensitive to our assumptions about the variance in energy consumption over time (ESM, figure B7). We did this by increasing the energy content of food items *b_j_* while decreasing their rate of discovery *γ*_G_ and *γ*_B_, such that the total amount of energy in the environment remained constant but the variance increased (implying longer periods without eating). The results are almost unchanged across the full range of the proportion of food that occurs under good conditions (ESM, figure B7).

**Figure 5.**
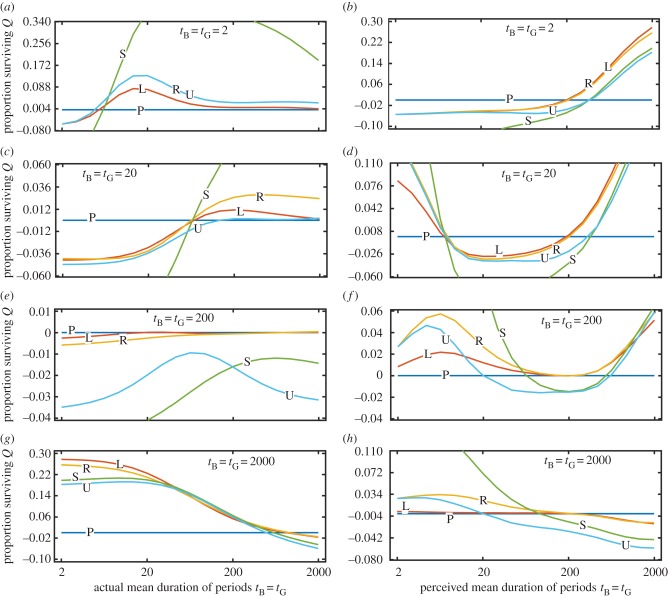
Probability of surviving 2000 time steps *Q(i)* when the actual fluctuation rates differ from those the animal is adapted to. Survival is plotted as the difference compared with survival under perfect knowledge (*P*); negative values imply lower survival as a result of imperfect knowledge about current conditions (different lines for strategies *L*, *R*, *S* and *U*). Left column (*a*,*c*,*e*,*g*) shows relative survival as a function of the actual mean duration of periods (*x*-axis) when following the optimal strategy for the duration shown on the panels. Right column (*b*,*d*,*f*,*h*) shows survival as a function of the mean duration of periods to which the animal is adapted (*x*-axis) in four environments with different actual mean durations (shown on the panels). (*a*,*b*) *t*_B_ = *t*_G_ = 2000; (*c*,*d*) *t*_B_ = *t*_G_ = 200; (*e*,*f*) *t*_B_ = *t*_G_ = 20; (*g*,*h*) *t*_B_ = *t*_G_ = 2. (Online version in colour.)

In addition to having imperfect knowledge about current conditions, a forager's perception of the pattern of environmental change may be prone to error. This may be the case because of dispersal or because anthropogenic change is altering environments faster than animals can adapt [26]. To investigate this, we assess the performance of the same five strategy classes in an environment that fluctuates on a different timescale from that to which the forager is adapted. In figure 5, we present the survivorship relative to the *P* case when the strategy is mismatched for the transition probabilities (for absolute values, see ESM, figure B8). Overall the survival of *P* is poorer than that of *R* and *L* if the perceived rate of environmental change is different to the actual rate. This occurs because the optimal decision depends on the forager's current state and its expectations about the future; if those expectations are wrong, then performance will be poor. This is ameliorated if the forager can adjust its expectations via learning or other changes in state, which are influenced by the real conditions. At the extreme, if the forager expects periods to be long then the performance of *P* worsens as the actual period durations decrease (ESM, figure B8), whereas performance improves for *L* and *R* (figure 5*g*)*.* If the actual duration of periods is much longer than expected, then it would be better to act as though conditions are always poor (*S*) (figure 5*d*,*f*,*h*), but there is always a range of perceived durations where *L* and *R* outperform *P.* When the expected durations are quite inaccurate, the actual durations determine whether *R* outperforms *L* or vice versa: if the actual durations are long, reserves become a reliable cue of current conditions (figure 5*f*,*h*), whereas if the actual durations are short, the Bayesian strategy performs better (figure 5*b*,*d*).

The maintained reserve level is similar under *L* and *R* but slightly shifted to lower reserves compared with *P* for baseline parameter values (ESM, figure B9). Storing a lower level of reserves is predicted across most of parameter space (ESM, figure B10), except where there is a small difference in food availability between good and bad conditions and conditions change slowly (ESM, figure B10b, f) or when conditions are more often good (ESM, figure B10d,h). Across all of parameter space, reserves under *L* are closer to those under *P* than *R*, explaining the slightly better performance of *L*.

## 4. Discussion

The need to track and respond appropriately to environmental conditions generates an important selective pressure on sensory and cognitive systems. Animals typically do not have perfect knowledge [27]. While foraging they may learn about the current food availability, but because food discovery is stochastic there is uncertainty. Given this uncertainty, animals are likely to have decision rules that perform well in most conditions [8,11,28]. The level of sophistication of these rules will depend on their associated costs and the benefit of tracking the environment. Here, we have compared the performance of a number of implementations of possible foraging mechanisms in an environment with fluctuating food availability. Our findings suggest that a Bayesian learning strategy—a commonly used paradigm in research on learning [6,9,12,29] but one which is arguably implausible for real organisms [6,7] (but see [9,10])—is unlikely to evolve under most conditions, because a simpler decision rule based solely on current energy reserves could allow the animal to perform almost as well. The greatest benefit to distinguishing between conditions occurs when the environment fluctuates slowly, but in this case there is ample time for energetic reserves to respond to current conditions before they change, and so most of the time the reserve level will be a sufficiently reliable indicator of current conditions. The ability to behave appropriately using only energy reserves as a cue is likely to have greatly reduced the selective pressure for sophisticated learning systems.

Bayesian learning might still be advantageous if other classes of strategy are very expensive, if there is a strong difference between conditions (making it more important to adjust behaviour accordingly) and if conditions change sufficiently fast that reserves are an unreliable cue to current conditions. This perspective suggests that animals in strongly and quickly fluctuating environments might be better at learning, which contradicts the suggestion that learning is favoured under intermediate rates of change [30,31]; note that these previous studies did not consider simpler alternative mechanisms. Strikingly, we predict that animals should be insensitive to some types of environmental fluctuations, such as if the fluctuations are not very large, or fluctuations are very quick, or if the world is usually in one state or the other. The latter result is predicted because if food conditions are dominated by one level of availability, then animals can just behave as though this is always the case. With fast changes or changes of small magnitude, it is less important to be sensitive to changes in food availability because current conditions do not provide much information about future conditions [20]. In experiments that have found no response to changing conditions [32], it is important to consider whether the study organism is adapted to an environment in which there is limited benefit of responding to changes.

In some situations, such as when the level of food availability changes frequently, it may be that the animal should do the same thing in the different conditions [20]. In such cases, an evolved mechanism may implement some simpler rule that does not try to track conditions (*U*). This may underlie state-dependent valuation of food sources, because an animal's state may reflect what conditions were generally like when particular sources were exploited [33].

The reserve-based strategy class may be the most likely evolutionary outcome in most situations. Even the simple rule (*U*) requires a basic sensitivity to reserve level to avoid starvation, and the reserve-based strategy is unlikely to involve significant additional costs. Thus, animals will not necessarily carry the level of reserves predicted by standard models that assume perfect knowledge or Bayesian learning, but instead may make systematic deviations because they are using reserves as a source of information. We predict that these deviations will be positive (more reserves than predicted by perfect information models) when conditions change slowly but negative when conditions change quickly (ESM). There may be no need for a cognitively encoded memory of recent foraging experiences; natural selection will simply exploit information by favouring an adaptive response to energetic reserves. In effect, the animal's reserves act as a physiological memory of past events. This suggestion could be tested empirically in systems where foraging experiences can be decoupled from the perceived level of reserves, for example, through experimental manipulation of hormones such as ghrelin and leptin that are involved in the regulation of feeding behaviour. By manipulating hormone levels and foraging experiences independently of each other, it should be possible to determine whether foraging behaviour is controlled by a cognitively encoded memory, a reserve-based memory or some combination of the two.

Lea *et al*. [34] assessed the performance of cognitive mechanisms for solving the explore-exploit trade-off. They found that a simple decision rule can perform better than more sophisticated strategies in some conditions, such as where there is insufficient time to learn about current conditions, which is comparable to the poor performance of our Bayesian learning strategy when fluctuations are frequent. However, the choice of foraging currency is likely to be crucial for the insights obtained [15], and often maximization of net rate as assumed by Lea *et al*. [34] will make substantially different predictions to currencies that incorporate the risk of mortality that most foragers face [15,35]. Future theoretical work should consider how a foraging rule based on physiological state, such as a hormone level, performs relative to a cognitive mechanism that attempts to learn about the level of predation risk from direct experiences (e.g. sightings of predators).

Learning rules that maximize long-term reward rate by learning about conditions can perform much better than ignorant rules [36,37]. But these rule sets have not accounted for the fact that internal state, such as the level of energy reserves or body temperature, always provides animals with some information and we expect natural selection to have formed strategies that exploit all sources of information about the external conditions. Several models have shown that an animal's state should influence decision-making to the extent that behaviour may appear irrational [38–41]. Here, we have identified that the effect of energetic reserves may be more complex still: animals with equal levels of reserves may differ in their response if they are adapted to different environments, such as different rates of change, because of how this affects the information content [20].

The marginal value theorem predicts that the marginal capture rate for leaving patches of prey should be higher when the overall prey abundance is higher, but this is often not observed [42]. A simple rule of thumb of a constant giving-up time results in behaviour that approximates the optimal solution much of the time [17,43,44]. Such a rule may be driven by some internal physiological state, involving feedback from the gustatory system, which reflects the time since the last prey item was consumed. Nonacs [45] showed that including a forager's energy reserves alters the predictions of the marginal value theorem, but he also assumed that animals could keep track of foraging success in a perfect way. We suggest that a better approach may be to model a gustatory state, such as stomach contents, which the animal can use as a cue of foraging success. Our reserve-based approach could be used to incorporate information constraints in many established models of animal behaviour and decision-making.

There is currently much interest and concern about the ability of organisms to cope with human-induced rapid environmental change [46]. Such rapid changes will cause there to be a mismatch between the conditions that animals have evolved to deal with and those they actually experience. Our results (figure 5) suggest that the details of how the environment has changed will determine how organisms respond. Interestingly, if environmental change causes conditions to fluctuate more quickly or more slowly than in the evolutionary past—for example, because it leads to more extreme weather patterns—then organisms that can perceive the current conditions directly (*P*) may in fact perform worse than those that use simple rules to estimate current conditions (figure 5). Which strategy class performs best depends on whether fluctuations are more or less frequent: if conditions now change more quickly than in the past, then learning does best (figure 5*b*,*d*), whereas if conditions change more slowly then simpler (e.g. reserve-based) strategy classes not based on learning do best (figure 5*f*,*h*).

We have shown that, in a foraging context, a behavioural strategy based only on an internal physiological state (*R*) can perform so well that more sophisticated strategies, such as learning directly from foraging outcomes (*L*) or accurately perceiving current conditions (*P*), might not provide sufficient advantages to offset their costs. It is striking that a reserve-based strategy is more robust to error in the pattern of environmental fluctuations than a rule based on perfect information about current food availability. Therefore, if the information about the environment is unreliable, we expect selection to favour simpler strategy classes. So far, we have been unable to prove that our methodology for finding the best-performing reserve-based strategy actually converges on the global optimum, rather than a local optimum (see ESM, figure B11). However, if it is just a local optimum, then our conclusions would be strengthened: the performance of the reserve-based strategy at its global optimum (elsewhere in *n*-dimensional space) would be even better than the one we have described here, and hence even closer to the performance of the Bayesian learning strategy.

Similar principles could well apply in other (non-foraging) contexts: any physiological or psychological state variable that is altered by experience might function as an efficient integrator (a ‘memory') of past experiences. An obvious candidate is emotions and moods, which have been modelled mechanistically [47] and may help an animal to adjust its behaviour adaptively when conditions are uncertain [48,49]. In fact, in non-foraging contexts, the state variable may have greater flexibility to act as a cue because (unlike energy reserves) the animal does not necessarily depend on it for survival, so it could potentially evolve to be more informative than energy reserves are in the foraging case. One intriguing possibility is that emotional states were initially unavoidable consequences of levels of neurotransmitter activity, but have been modified by selection to provide more reliable information about recent experiences and thereby influence cognitive decisions. If the principle we have highlighted applies to most physiological states, then organisms may often appear to be cognitively sophisticated despite basing their decisions on relatively simple mechanisms. Since internal states can summarize a great deal of information about the environmental conditions, they will reduce the selective pressure to learn directly from the immediate outcomes of decisions. Animals are therefore likely to be cognitively unsophisticated when they are able to perform well using simple mechanisms.

## Supplementary Material

Online Appendices

## Data accessibility

The code used to generate the results have been uploaded as part of the ESM.
