# Supplementary material for: Trust your gut: using physiological states as a source of information is almost as effective as optimal Bayesian learning
Source: Proc Biol Sci. 2018 Jan 24;285(1871):20172411. doi: 10.1098/rspb.2017.2411 (PMC5805941; doi:10.1098/rspb.2017.2411)

Online Appendices

**Trust your gut: using physiological states as a source of information**

**is almost as effective as optimal Bayesian learning**

A. D. Higginson, Tim W. Fawcett, Alasdair I. Houston, John M. McNamara

**APPENDIX A: Implementation of the model**

Here, we provide a complete description of the model implementation. First, we describe the method for finding the optimal strategy under perfect information (following [1]) and then describe how this process is modified to find the other strategies. Our optimization criterion follows that of [2]. At a decision epoch reserves are assumed to take values in the range $x=0, 1, 2, \cdots,s$, and the environmental conditions *E* can be either good (*E* = G) or bad (*E* = B). At each decision epoch the animal chooses the intensity of foraging, *f*. Under the perfect information case, *f* is allowed to depend on both the energy reserves of the animal and the current environmental conditions. A strategy $\pi$ specifies this dependence; under $\pi$ the proportion of time foraging when reserves are *x* and the environmental conditions are *E* is denoted by$f_{\pi}(x,E)$. Let $S_{\pi}(x,E,t)$ be the probability that an animal that follows strategy$\pi$ survives until time *t* given that its reserves are *x* and the environmental conditions are *E* at time 0. Then it follows by standard Markov chain results that there exists a function of state $h_{\pi}(x,E)$and a constant $\alpha(\pi)$ such that

$S_{\pi}(x,E,t) \sim h_{\pi}(x,E)(\alpha(\pi))^{t}\text{ as }t\to\infty$. (A1)

The function *h* represents the transitory effect of initial conditions. The constant $\alpha(\pi)$ is the probability that an animal that is alive after some long time *t* is still alive at *t*+1. Thus, if$\alpha(\pi_{1})<\alpha(\pi_{2})$ for two strategies$\pi_{1}\text{ and }\pi_{2}$, the probability of surviving until time *t* is greater under $\pi_{2}$ than under $\pi_{1}$ for all sufficiently large *t*; i.e. $S_{\pi_{2}}(x,E,t)>S_{\pi_{1}}(x,E,t)$ for all sufficiently large *t*. Motivated by this we define a strategy $\pi*$ to be optimal if

$\alpha(\pi*)=\max_{\pi} \alpha(\pi)$. (A2)

We assume that the chance of finding food increases linearly with foraging intensity. Thus, if the animal forages at intensity *f* between time *t* and *t* + 1, then the probability of finding an item of food during this time interval is *fγ_E_*. Wwe assume that food items are of two types (type *j* = 1, 2) with relative abundance *ϕ_j_* and provide a reward of energetic value *b_j_*. This avoids potentially problematic grid effects that can result in discontinuities in the decision arrays, which would be unrealistic: the decisions of real animals typically respond smoothly to small changes in influential variables (for further discussion, see [3], p. 35).

We assume that the forager pays an energy cost *m* to meet its metabolic needs in each time step. The probability the animal is not killed by a predator before the next decision epoch is $1-\mu(x,f)$. Thus if the animal has reserves *x* at time *t* and forages with intensity *f* its reserves at time *t* + 1 are either *x-m*, *x + b_1_ – m* or *x + b_2_ – m*, and these possibilities occur with probabilities $[1-\mu(x,f)][1-f\gamma_{E}]$, $[1-\mu(x,f)]f\gamma_{E}\varphi_{1}$, and $[1-\mu(x,f)]f\gamma_{E}\varphi_{2}$, respectively. If the change in reserves results in the new reserves being zero or below, the animal is assumed to have died of starvation. If the new reserves would have been greater than the maximum value *s*, then reserves are taken to be *s*.

Given these ingredients, the dynamic programming operator *T** can be expressed follows. Let *V* be a function, $V\left(x,E\right)$, of energy reserves *x* and environmental conditions *E* satisfying $V\left(0,E\right)=0$. *V* gives the relative probability of survival for any long period under an optimal strategy. This probability is relative in the sense that the ratio of the probability of survival from state (*x*_1_, *E*_1_) compared to that from state (*x*_2_, *E*_2_) is *V*(*x*_1_, *E*_1_)/*V*(*x*_2_, *E*_2_). The rescaling in our computation leaves these relative values unchanged. *V* is proportional to the expected future reproductive success.

$T *V$is a new function of reserves and environmental conditions that satisfies $(T *V)\left(0,E\right)=0$for all *E* and$(T*V)\left(x,E\right)=\max_{f} H(x,E;f,V)$ for *x* > 0 and all *E*, where

$H(x,E;f,V)=\left[1-\mu\left(x,f\right)\right]\left(\begin{matrix} \left(1-\gamma_{E}f\right)\left[\left(1-\lambda_{E}\right)\cdot V\left(\max(x-m,0),E\right)+\lambda_{E}\cdot V\left(\max(x-m,0),\neg E\right)\right] \\ +\gamma_{E}f\left[\left(1-\lambda_{E}\right)\sum_{j=1}^{2} \varphi_{j}\cdot V\left(\min\left(x+b_{j}-m,s\right),E\right)+\lambda_{E}\sum_{j=1}^{2} \varphi_{j}\cdot V\left(\min\left(x+b_{j}-m,s\right),\neg E\right)\right] \end{matrix}\right)$(A3)

where *¬E* indicates the other value of *E*. Here, the first term represents the probability of surviving to the next time step; the first term in large brackets is the probability of failing to find food multiplied by the resulting value function in the next time step (with lower limit of zero); the second term in brackets is the probability of finding food multiplied by the resulting value function in the next time step (with upper limit of *s*). Hence, the function *H* gives the relative probability of survival if the animal takes decision *f* in state (*x, E*), under the assumption that its subsequent behaviour is optimal.

To find the optimal strategy, we define a sequence of functions $V_{0}, V_{1}, V_{2},\cdots$ iteratively as follows. Initially set $V_{0}\left(0,E\right)=0$ for all *E* and $V_{0}\left(x,E\right)=1$ for all $x>0$ and all *E*. Given $V_{k}$, set $V_{k+1}=T *V_{k}/\left|T *V_{k}\right|$, where the norm, $\left|V\right|$, of a function *V* is given by $\left|V\right|=\max_{x,E} \left|V(x,E)\right|$. Then the sequence of functions converges pointwise to a limit $V *=\lim_{k\to\infty} V_{k}$ [2]. Convergence was judged to have occurred when $\left|V_{k+1}-V_{k}\right|<10^{-6}$, which typically happened within 500 iterations. Any strategy $\pi*$ satisfying

$H(x,E;f_{\pi*}(x,E),V*)=\max_{f} H(x,E;f,V*)\text{ for all }x>0\text{ and all }E$ (A4)

necessarily satisfies equation (A2), and is hence optimal [2]. For strategy *P* (perfect information) we define $f_{P}(x,E)=f_{\pi}(x,E)$for all *x* and *E*. All computations are based on the values $b_{1}=5$, $b_{2}=6$, $\varphi_{1}=0.5$, $\varphi_{2}=0.5$ (so that the mean energetic value of a food item is 5.5) and *m* = 1.

**Constrained strategies**

The method outlined above assumes that the animal can perceive the current environmental state *E* directly. If this is not the case, it may be able to estimate *E* based on other available information. We therefore include a state variable *ρ* to represent the estimated probability that conditions are currently good (i.e. that *E* = *G*). For an animal with estimate *ρ* and current reserve level *x*, the optimal foraging intensity *f** is that which maximises the weighted sum

$$\rho\cdot W(x,\rho,G;f,V)+(1-\rho)\cdot W(x,\rho,B;f,V)$$

where *W*(*x*,*ρ*,*E*;*f,V*) is the relative probability of survival if the animal takes decision *f* in state (*x, E*) when the value function one unit of time later is *V*, the true environmental state is *E* and the animal’s estimate that *E* = *G* is *ρ*. *W* is given by

$W(x,\rho,E;f,V)=\left[1-\mu\left(x,f\right)\right]\left(\begin{matrix} \left(1-\gamma_{E}f\right)\left[\left(1-\lambda_{E}\right)\cdot V\left(\max(x-m,0),\rho_{t+1},E\right)+\lambda_{E}\cdot V\left(\max(x-m,0),\rho_{t+1},\neg E\right)\right] \\ +\gamma_{E}f\left[\left(1-\lambda_{E}\right)\sum_{j=1}^{2} \cdot\varphi_{j}V\left(\min\left(x+b_{j}-m,s\right),\rho_{t+1},E\right)+\lambda_{E}\sum_{j=1}^{2} \cdot\varphi_{j}V\left(\min\left(x+b_{j}-m,s\right),\rho_{t+1,}\neg E\right)\right] \end{matrix}\right)$ We consider three possible constrained strategies that differ in how they estimate the environmental conditions: (i) a Bayesian learning strategy (*L*) that updates *ρ* based on its past experience of successes and failures in foraging, using Bayes’ rule; (ii) a reserve-based strategy (*R*) that estimates *ρ* based solely on its current reserve level; and (iii) a pessimistic strategy *S* that behaves as though *ρ* = 0. Below we explain how *f** is computed in each case.

**Bayesian Learning (*L*)**

The initial prior estimate before any experience is gained (at *t*=0) is the long-term probability that the world is good:

$\rho_{0}=\frac{\lambda_{B}}{\lambda_{B}+\lambda_{G}}$.

At each time step this estimate is revised on the basis of the animal’s experiences, using Bayes’ rule. Before the animal acts the conditions may change, so the updated prior in time step *t* is

$\hat{\rho}_{t}=(1-\rho_{t})\lambda_{B}+\rho_{t}\lambda_{G}$.

If the animal chooses to forage, it experiences either a failure or a success. The posterior probability that conditions are good at the start of the next time step, *ρ_t_*_+1_, is given by Bayes’ rule:

.

These expressions allow us to update *ρ* iteratively from one time step to the next. To identify the optimal strategy, we discretise *ρ* (into 101 values 0, 0.01, 0.02 … 1.0) and find the foraging intensity that maximises $\rho\cdot W(x,\rho,G;f)+(1-\rho)\cdot W(x,\rho,B;f)$ for a two-dimensional matrix of (*x,ρ*), following the method described above and in [2]. We consider the strategy at convergence to be the optimal Bayesian strategy, which we denote by *f*_L_*(*x*,ρ).

**Reserve-based inference (*R*)**

We now consider strategy that does not learn but uses its current reserve level as a source of information. Such a strategy could evolve based on the historic relationship between conditions and reserves, which would be encoded in the genotype over evolutionary time or developed during ontogeny by long-term learning. The response to the reserve level should be adjusted to the long-term probability of different conditions given the reserve level, because reserves (which can be directly perceived by the animal) are correlated with food availability (which cannot be directly perceived). In practice, this involves considering *ρ*(*E|x*), the probability of environmental conditions *E* given the energetic reserves *x*, where

$\sum_{E\in\{B,G\}} \rho(E|x)=1\text{ }$ for all *x*.

The animal’s decision at each level of reserves is influenced by a weighted sum of its possible future values (in good and bad conditions), but here the weighting is the array *ρ*(*E|x*). The constrained optimal reserve-based inference strategy, which we denote by *f*_R_*(*x*), is the vector of *f* that maximises

$\sum_{E\in\{B,G\}} \rho(E|x)\cdot W(x,\rho,E;f,V)$.

Note that *ρ*(*E|x*) depends not only on environmental conditions (*λ_E_* and *γ_E_*) but on the strategy *f*_R_*(*x*). Therefore an additional iterative process is required to find *ρ*(*E|x*). By Markov chains, we find the stationary distribution of reserve levels (*x*) under good conditions (*E* = *G*) and under bad conditions (*E* = *B*), in a very large (infinite) population of individuals following the optimal strategy. This procedure is as follows. We initialise the population with an equal frequency of individuals, *N(x,E*), in all states. There are *s* reserve levels from *x* = 1 to *x* = *s* and two environmental states, so the frequency of each state is initialised as:

$N_{i}(x,E)=\frac{1}{2s}\text{ }$ for all (*x,E*).

where the denominator is the number of "alive" states in the matrix *N_i_* (i.e. excluding *s*=0, in which case the animal has starved to death). Given *f**(*x*) and the probabilities of each event (conditions changing, finding food, being killed by a predator) we can calculate the corresponding frequencies one time step later, *N_i+_*_1_(*x,E*). We iterate this process, rescaling each time step so that

$\sum_{x=1}^{s} \sum_{E\in\{G,B\}} N_{i+1}(x,E)=1$.

Note that we omit individuals that have been killed or have starved (*x*=0). At convergence (judged to have occurred when $\left|N_{i+1}-N_{i}\right|<10^{-4}$), *N* is the probability distribution of a large population of survivors across the two states, which we denote $\hat{N}$. We then use this to update *ρ*:

and .

We then perform the backwards recursion again to find *f*_R_*(*x*) given the new *ρ*(*E|x*). We iterate this process until $\rho(G|x)$changes by less than 10^-4^.

**Pessimist (*S*)**

The pessimistic strategy assumes that *ρ* = 0 for all time steps (i.e. it behaves as though conditions are always bad).

**Optimist**

The optimist strategy assumes that *ρ* = 1 for all time steps (i.e. it behaves as though conditions are always bad). Performance was so poor that results are not presented, but the available Matlab code runs this strategy.

**Optimal bias (*U*)**

The optimal bias strategy assumes that *ρ* takes an optimal value *ρ** for all time steps given that the animal behaves as though there is no autocorrelation in environmental conditions (i.e. it behaves as though conditions are currently bad with probability *ρ**). We calculate the long-term mortality rate (see below) of optimal strategies based on 101 values of *ρ* between 0 and 1, and then take the value which leads to greatest survivorship, which we call *ρ**.

**Calculating survival**

For all classes of strategy, the probability that the forager survives an arbitrarily long period can be calculated by forwards iteration. We do this by simulating a population following the optimal strategy until the distribution of individuals stops changing, rescale so the size of the population is unity, and then assess survival over an additional 2000 time steps. This gives the survival probability *Q*(*i*).

**References**

1. Higginson, A. D., Fawcett, T. W., Trimmer, P. C., McNamara, J. M. & Houston, A. I. 2012 Generalized optimal risk allocation: foraging and antipredator behavior in a fluctuating environment. *Am. Nat.* **180**, 589–603. (doi:10.1086/667885)

2. McNamara, J. M. 1990 The policy which maximises long-term survival of an animal faced with the risks of starvation and predation. *Adv. Appl. Probab.* **22**, 295–308.

3. Houston, A. I. & McNamara, J. M. 1999 *Models of adaptive behaviour*. Cambridge, U.K.: Cambridge University Press.

**APPENDIX B: Supplementary Results**

Figure B1: Probability that conditions are good given reserves under strategy *R, ρ*(*G|x*) for a range of conditions shown above panels. The values on each line indicate (a) the difference between conditions in food availability *γ_G_ – γ_B_*, when *γ_G_+γ_B_*=1 (b) *t_B_* and *t_G_* (c) *t_B_* with *t_G_* fixed at 100 (d) *t_G_* with *t_B_* fixed at 100. Values of parameters that are the same for all lines are indicated above panels.


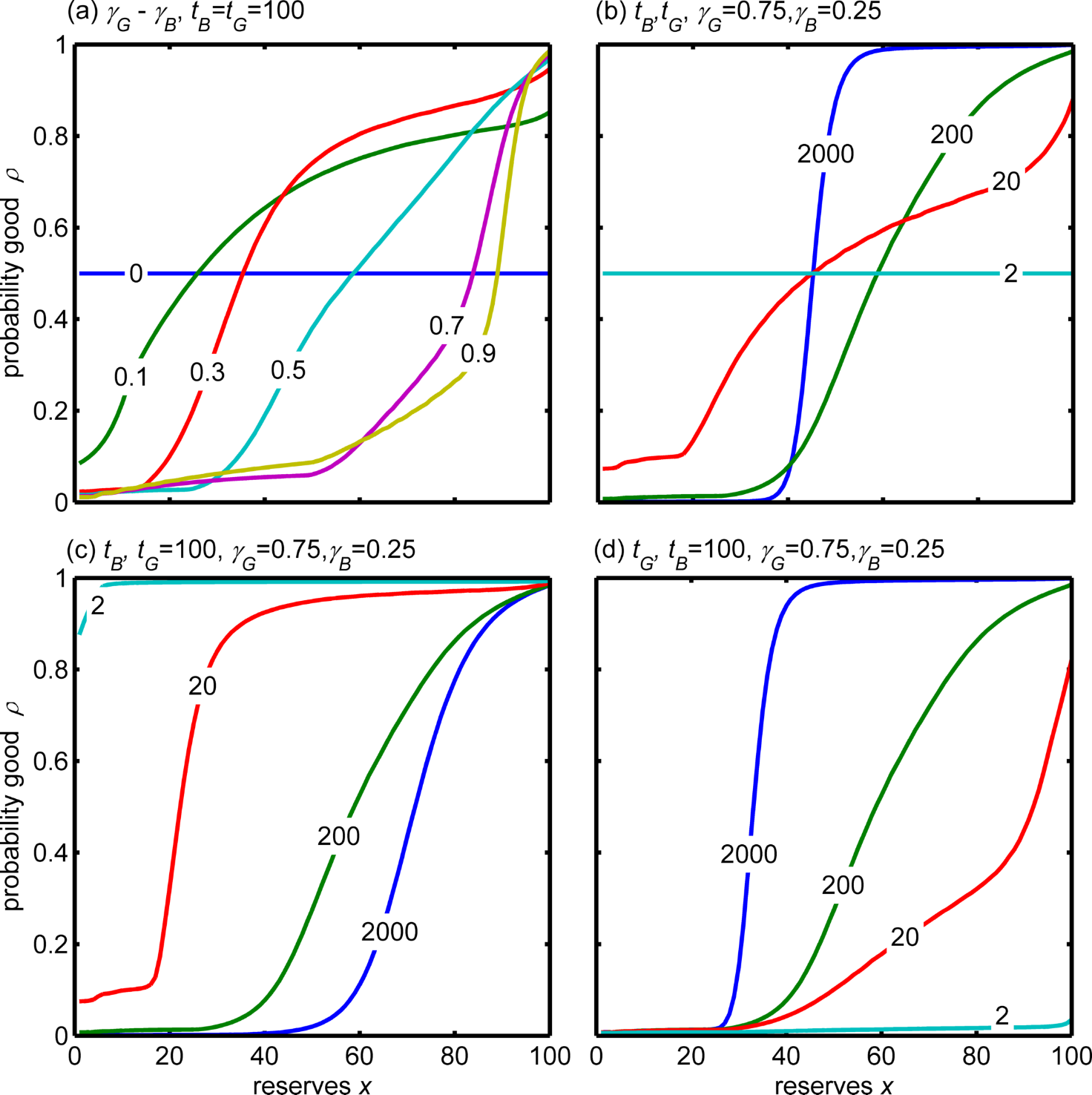


Figure B2: (a) The optimal decisions when: *E* is known (grey lines: *P_G_* solid, *P_B_* dashed); reserve-based (*R*), pessimist (*S*), optimal bias (*U*), for the baseline parameter values (Table 1). (b, c) The difference in foraging rate between each mechanism (labels as above) and the foraging rate when *E* is known for (b) bad and (c) good conditions.





Figure B3: Optimal Bayesian strategy *f*_L_* for the baseline parameter values (yellow *f*=1, red *f*=0).


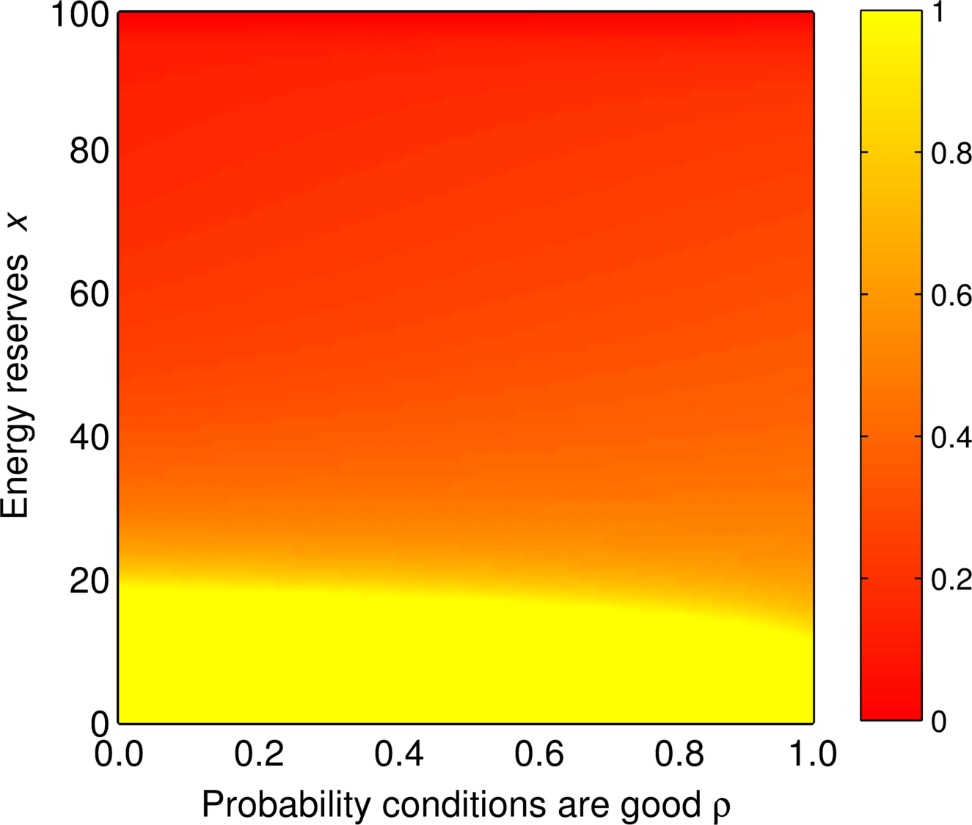


**
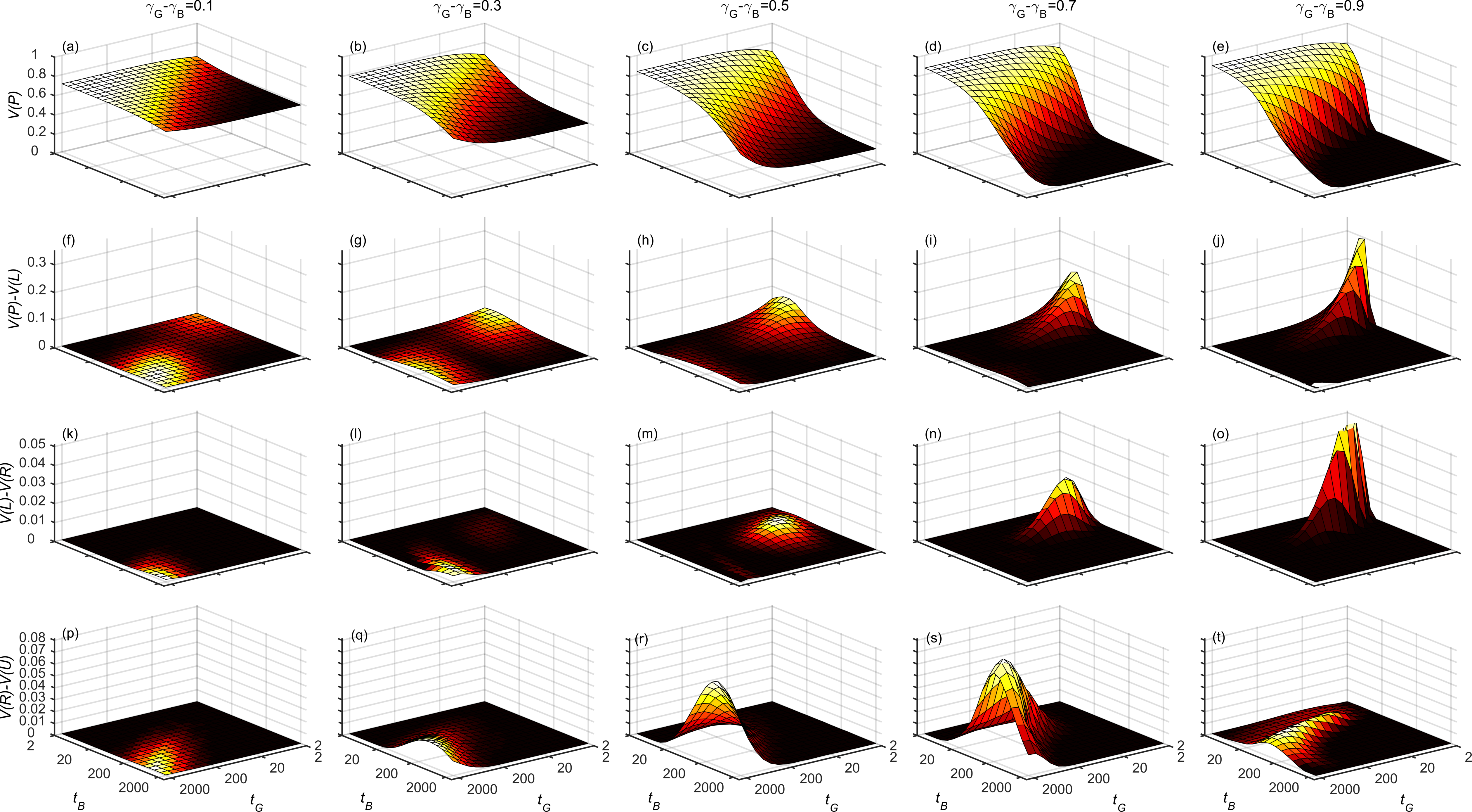
Figure B4**: Comparison of survival probabilities over 2000 time steps *Q(i)* for the various methods across parameter space of the mean duration of periods (*t_B_* and *t_G_* shown on axes). We show results for the difference in the probability of finding food between conditions (left-to-right shown at top of column): first column *γ_B_*=0.45, *γ_G_*=0.55; second column *γ_B_*=0.35, *γ_G_*=0.65; third column *γ_B_*=0.25, *γ_G_*=0.75; fourth column *γ_B_*=0.15, *γ_G_*=0.85; fifth column *γ_B_*=0.05, *γ_G_*=0.95. The top row (a–e) shows survival under perfect information (*P*) and the following rows show differences in survival between strategies (f–j) *P* and *L*: Bayesian learner; (k–o) *L* and *R*:reserve-based; (p–t) *R* and *U*:optimal bias. Note the different scales of the vertical axes.

**Figure B5**: Optimal *ρ* for strategy *U*. (a) Probability of surviving 2000 time steps for 101 (0, 0.01,…1) possible values of the subjective probability of high food conditions (*ρ**) and the survival under the other strategies (shown on horizontal lines), for the baseline parameter values. Under these conditions, where the real *ρ* is 0.5, optimal *ρ** is 0.22. (b, c, d) *ρ** for a range of the mean duration of periods (x-axes): (b) *t_B_* and *t_G_* (c) *t_G_* with *t_B_* fixed at 100 (d) *t_B_* with *t_G_* fixed at 100. The values on each line indicate the difference between conditions in food availability *γ_G_ – γ_B_* (values as in Figure B4).

*
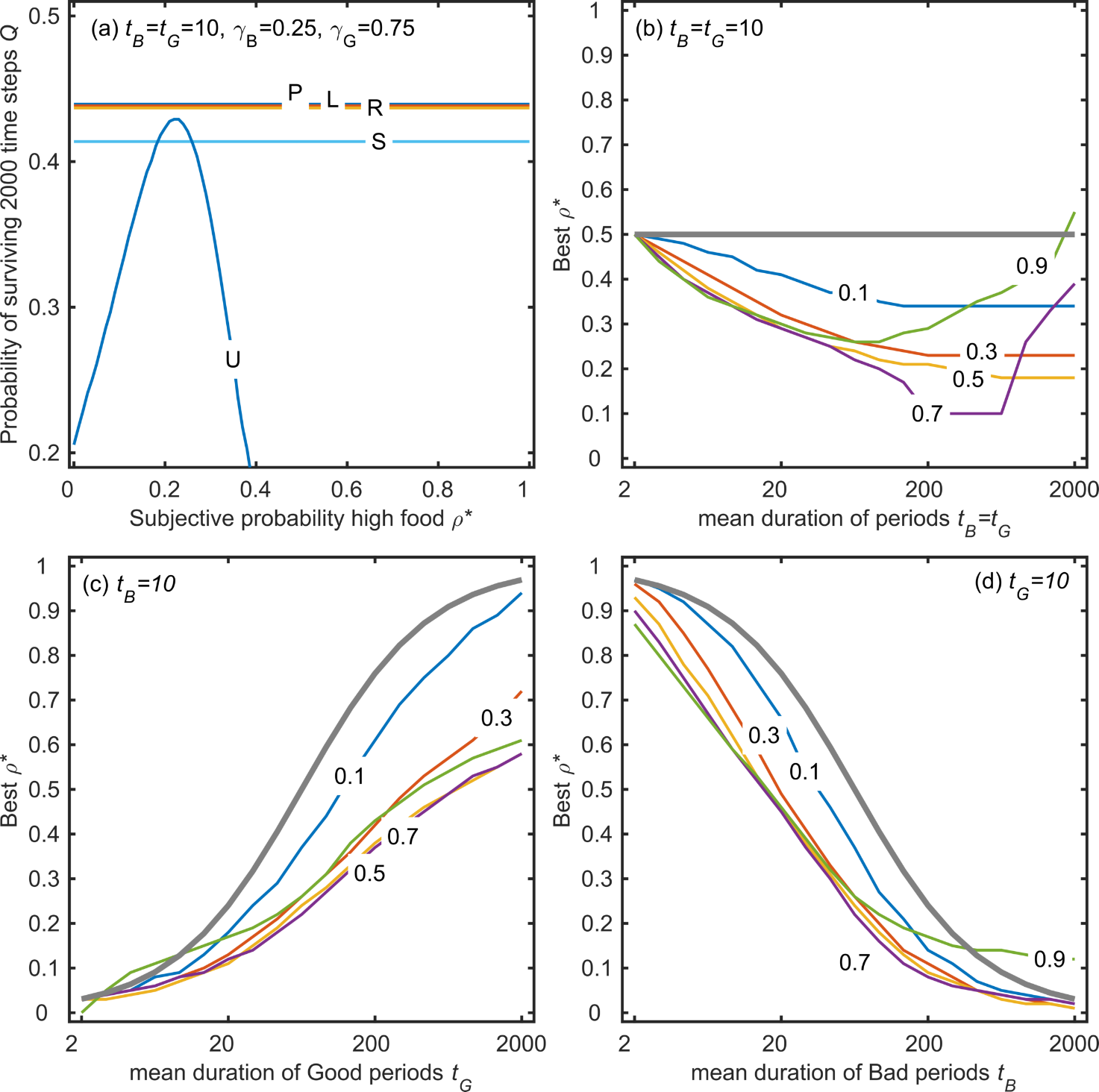
*

**Figure B6**: Comparison of survival probabilities over 2000 time steps *Q(i)* for the various methods across parameter space of the probabilities of finding food (*γ_B_* and *γ_G_* shown on axes). We show results for the mean duration of good and bad conditions (left-to-right shown at top of column): first column *t_B_*=*t_G_*=2000; second column *t_B_*=*t_G_*=200; third column *t_B_*=*t_G_*=20; fourth column *t_B_*=*t_G_*=2. The top row (a–d) shows survival under perfect information (*P*) and the following rows show differences in survival between strategies (e–h) *P* and *L* (Bayesian learner); (i–l) *L* and *R* (reserve-based); (m–p) *R* and *U* (optimal bias). Note the different scales of the vertical axes.


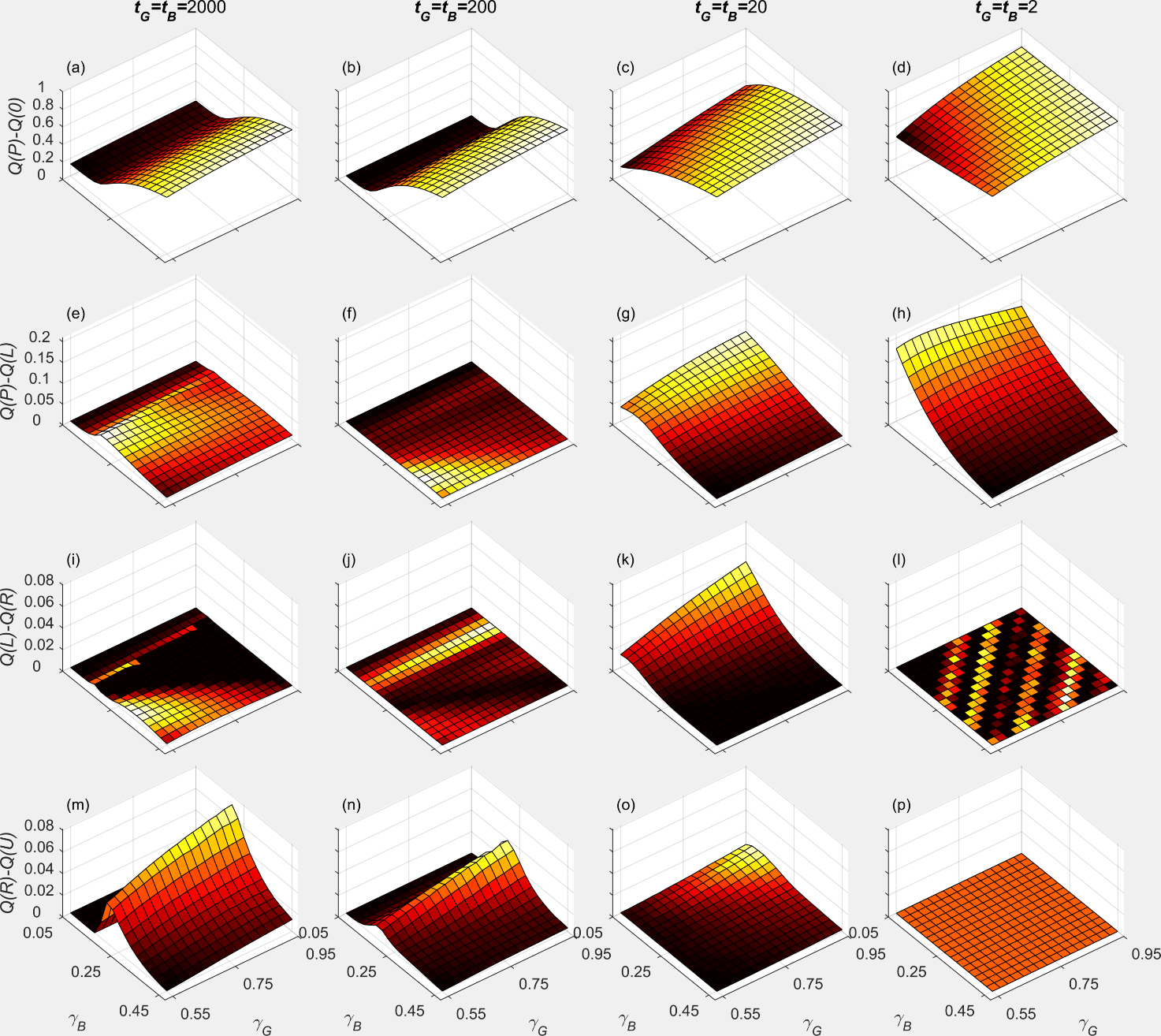


**Figure B7**: Effect of proportion of food in good conditions (x-axes) on difference in survival from survival under perfect knowledge of the optimal Bayesian learning strategy (*L*), the reserve-based case (*R*), and the optimal bias (*U*) for four sizes of the two food items. Panel (a) is the food size *b_j_* and availability *γ_E_* in the main text. In other panels we increase *b_j_* (as shown) and decrease γ*_B_* and γ*_B_* such that γ*_B_* + γ*_B_* = 5.5 / [(*b_1_* + *b_2_* )/2], meaning that the total amount of energy in the environment was constant for all value combinations. The results are almost unchanged for the range of the proportion of food that is in the good condition (x-axes).


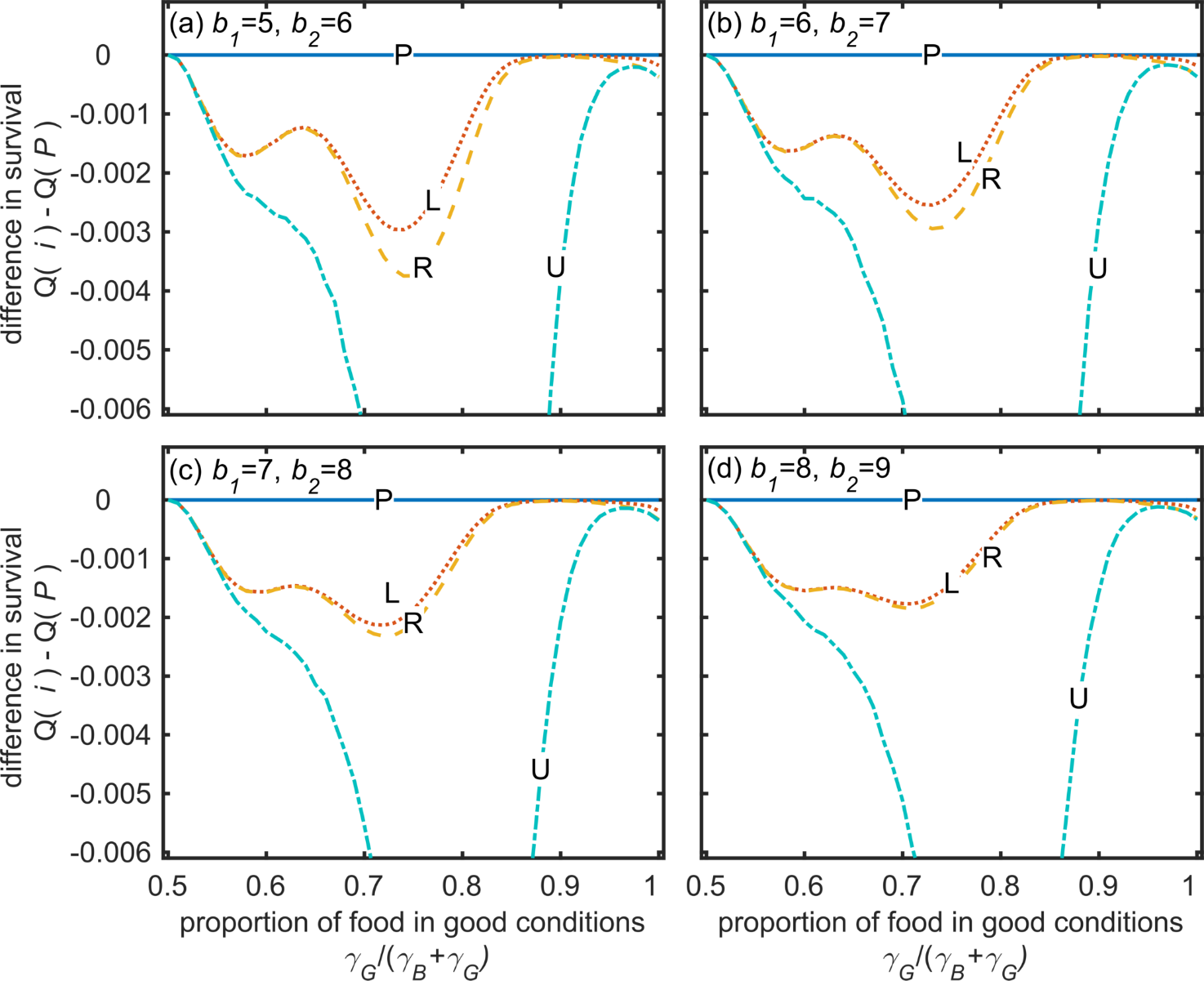


**Figure B8:** Probability of surviving 2000 time steps *Q(i)* when the actual fluctuation rates differ from those the animal is adapted to. Left column (a, c, e, g) shows survival as a function of the actual mean duration of periods (x-axis) when following the optimal strategy for the duration shown on the panels. Right column (b, d, f, h) shows survival as a function of the mean duration of periods to which the animal is adapted (x-axis) in four environments with different actual mean durations (shown on the panels); note that survival is maximised for when the actual and adapted-to durations match. (a, b) *t_B_*=*t_G_*=2; (c, d) *t_B_*=*t_G_*=20; (e, f) *t_B_*=*t_G_*=200; (g, h) *t_B_*=*t_G_*=2000.

**
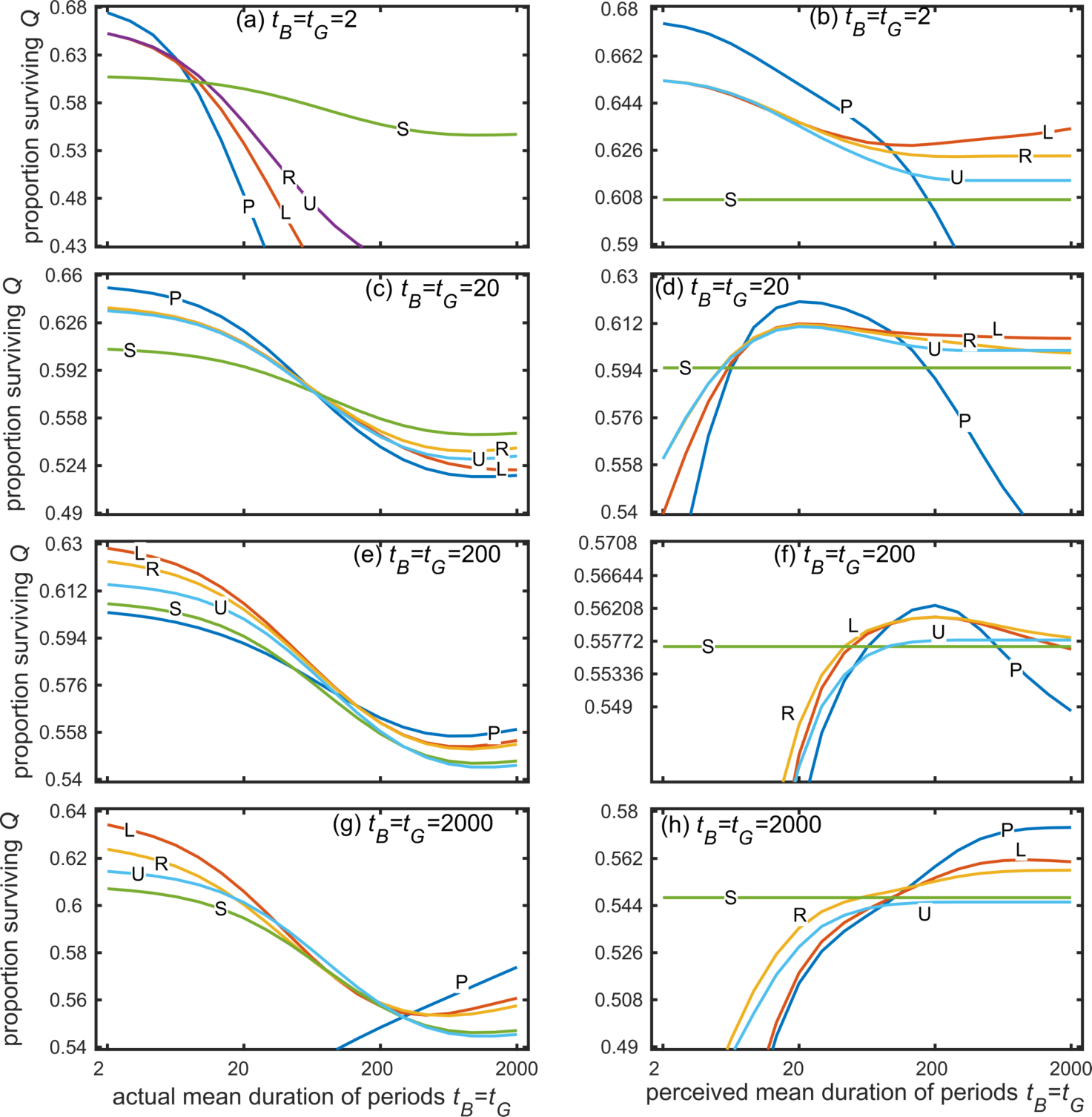
**

**Figure B9**: Stationary distribution of reserves in (a) bad and (b) good conditions for each strategy (not showing dead individuals) and difference to *P* under (c) bad and (d) good conditions, for the baseline parameter values (Table 1). Note how similar reserves are under *R* and *L*.


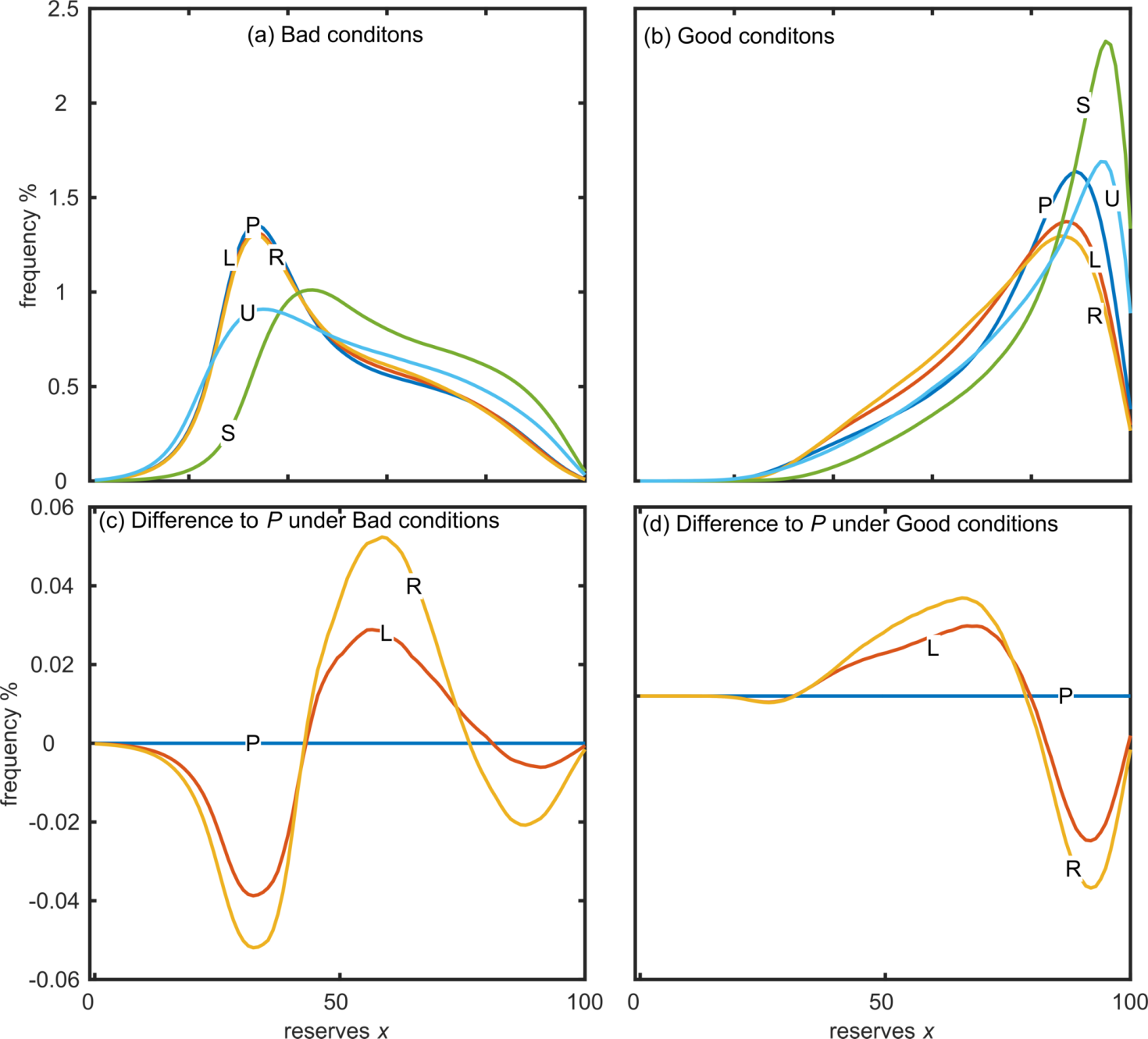


**Figure B10**: (a-d) Mean reserves and (e-f) difference in mean reserves between *P* and *R* and *L* for surviving individuals averaged across both conditions at the stationary frequency distribution, in relation to (a, b, e, f) the difference in the probability of food between conditions when both good and bad periods (a, e) are moderately short and (b, f) moderately long, and (c, g) the mean duration of bad periods, and (d, h) the mean duration of good periods.


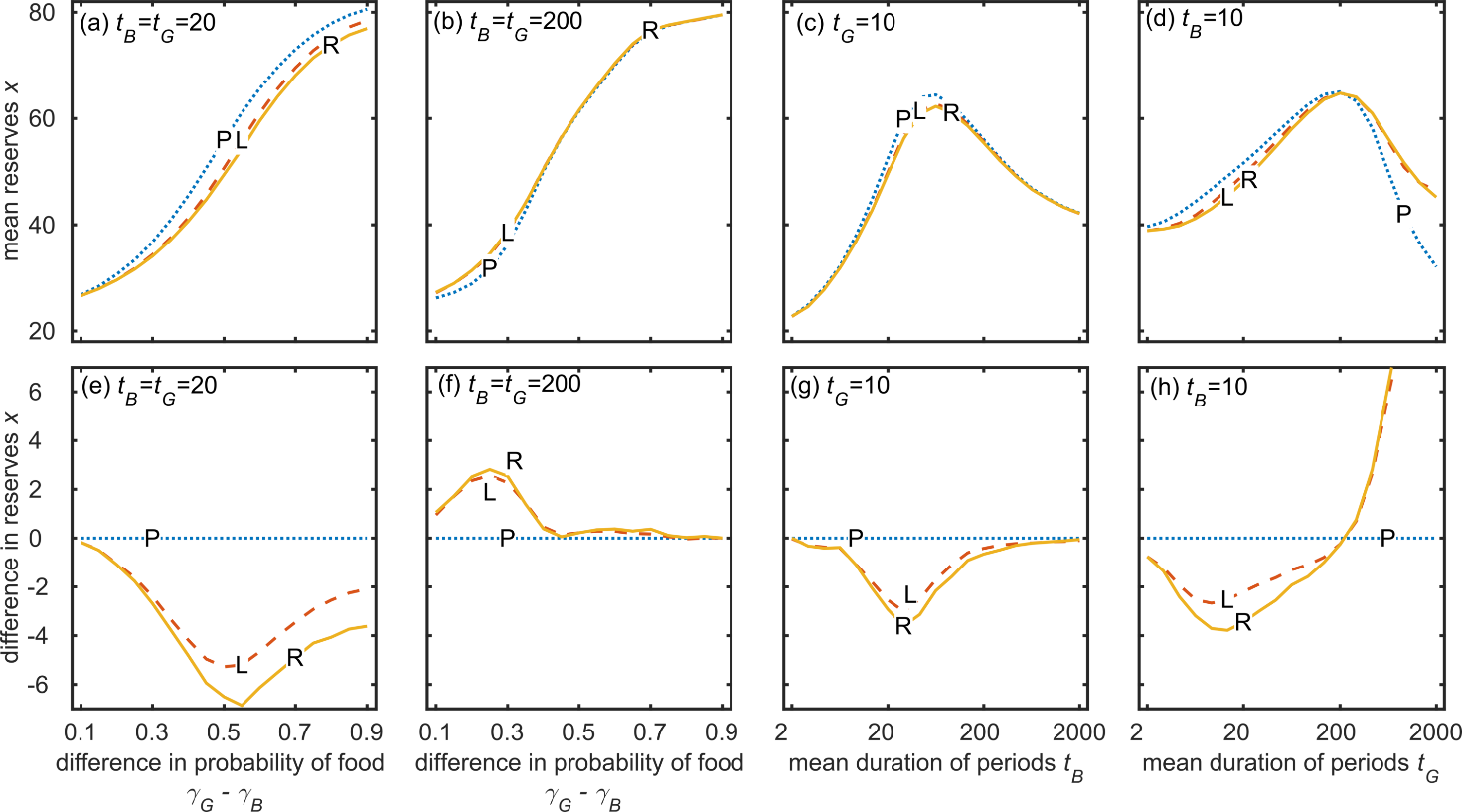


**Figure B11:** Effect of deviation from the optimal strategy on the probability of surviving 2000 time steps. We calculate survival when one value of *f*_R_* was changed from the optimal value by the amount shown on the x-axis. The value changed was for the value of *x* shown on the lines. In all cases survival was reduced from the survival under the optimal strategy, and as the absolute change increased. The reduction in survival was greatest for the *x* values which are maintained by the animal (Figure B9, 30<*x*<50). In sum, this indicates that the procedure finds at least a local optimum, which may also be the global optimum.


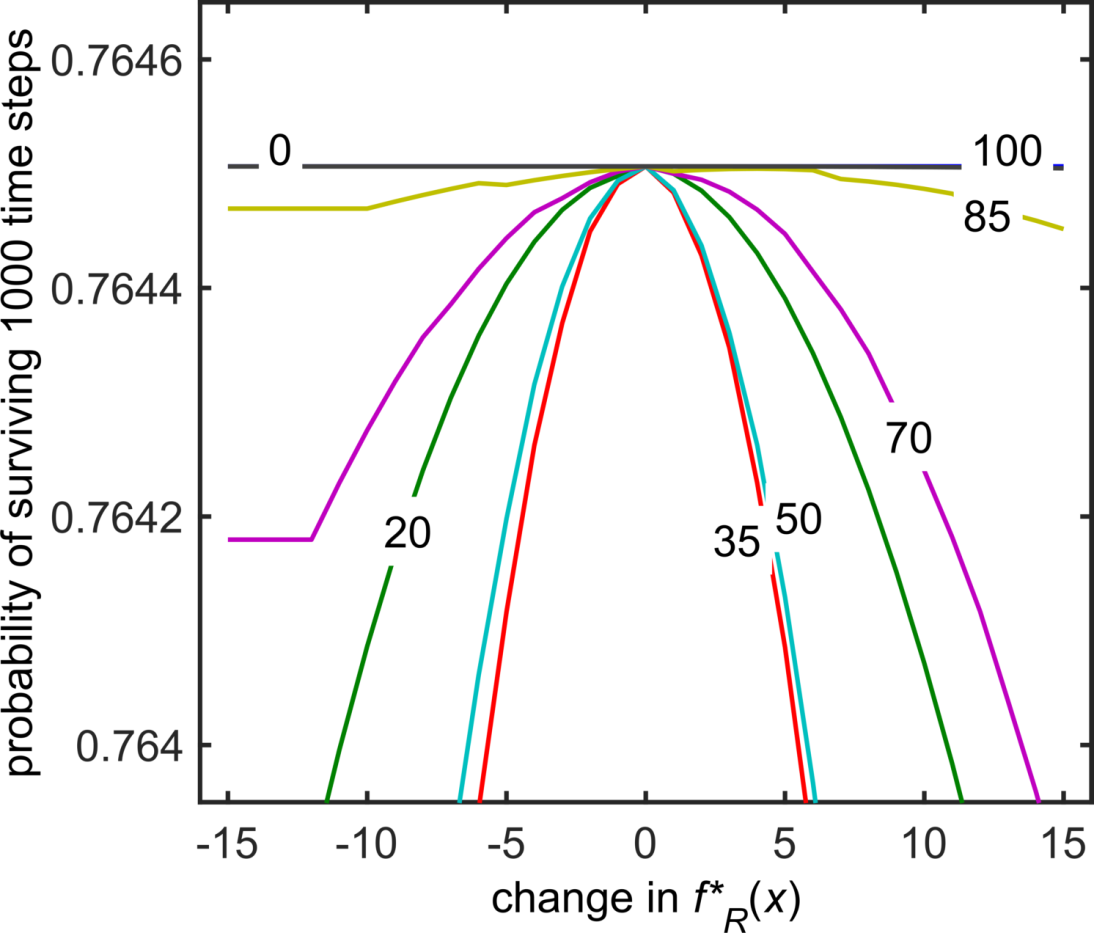

Supplement: Online Appendices [file rspb20172411supp1.docx]
